# Median effective dose of intravenous nalmefene for preventing sufentanil-induced cough in children aged 1–6 years: a prospective randomized controlled trial

**DOI:** 10.3389/fped.2026.1833973

**Published:** 2026-07-20

**Authors:** Chao Wang, Fan Wang, Lijing Li, Xiaoxue Wang, Lanxin Qiao, Fang Wang

**Affiliations:** Department of Anesthesiology, Beijing Children’s Hospital, Capital Medical University, National Center for Children’s Health, Beijing, China

**Keywords:** anesthesia induction, dose-response relationship, median effective dose, nalmefene, pediatric anesthesia, randomized controlled trial, sufentanil-induced cough, **μ**-opioid receptor antagonist

## Abstract

**Background:**

Sufentanil-induced cough (SIC) during anesthetic induction carries greater risks in young children than in adults owing to their limited functional residual capacity and elevated oxygen demand. No optimal prophylactic dose of intravenous nalmefene has been established for this age group. We aimed to determine the median effective dose (ED50) of nalmefene for preventing SIC in children aged 1–6 years.

**Methods:**

In this prospective, randomized, double-blind, placebo-controlled trial (ChiCTR2500111207), 249 children aged 1–6 years undergoing elective laparoscopic surgery were assigned 1:1:1:1 to normal saline (Group A) or nalmefene 0.025, 0.05, or 0.1 μg/kg (Groups B, C, and D), each administered one minute before sufentanil 0.2 μg/kg. The primary endpoint was SIC incidence within one minute of sufentanil injection. The ED50 was estimated using a three-parameter sigmoidal Emax model with bootstrap confidence intervals.

**Results:**

SIC incidence declined dose-dependently: 45.9% (Group A), 38.1% (Group B), 30.2% (Group C), and 11.3% (Group D). Groups A vs. D and B vs. D differed significantly (both *P* < 0.05, Bonferroni-corrected). Severe cough was eliminated in Group D versus 16.4% in Group A (*P* < 0.05). No between-group differences were detected in hemodynamic parameters or postoperative FLACC scores (*P* = 0.267). The ED50 was 0.064 μg/kg (95% CI: 0.036–0.096 μg/kg); the estimated ED95 was 0.242 μg/kg (95% CI: 0.114–1.948 μg/kg), requiring extrapolation beyond the observed dose range.

**Conclusions:**

Intravenous nalmefene prevents SIC dose-dependently in children aged 1–6 years. The ED50 of 0.064 μg/kg provides a clinically applicable reference dose that preserves hemodynamic stability and postoperative analgesia.

**Trial registration:**

Chinese Clinical Trial Registry, ChiCTR2500111207 (prospectively registered 25 February 2025).

## Background

Opioid analgesics, particularly sufentanil, are integral to anesthetic induction in pediatric surgery, providing rapid onset, potent analgesia, and cardiovascular stability. However, intravenous bolus administration of sufentanil is frequently complicated by cough—an adverse effect with a reported incidence of 15%–47% in clinical practice (1, 2). Although SIC is generally considered a minor inconvenience in adults, its consequences are substantially more serious in young children.

Young children have higher metabolic oxygen consumption per unit body weight and a proportionally smaller functional residual capacity than adults, leaving a narrow safety margin during apnea (3, 4). An episode of explosive coughing and associated breath-holding further reduces the limited pre-oxygenation window, increasing the risk of rapid hemoglobin desaturation (1). Additionally, the mechanical force of coughing may precipitate swelling of the tongue and hypopharynx, potentially causing acute airway obstruction (5). In patients with pre-existing cerebral aneurysm, open-eye injury, or pneumothorax, cough-mediated elevations in intracranial, intraocular, and intra-abdominal pressure can be particularly hazardous.

Several pharmacological strategies have been proposed to prevent SIC, including pretreatment with dezocine (6), nalbuphine (7), remifentanil (8), dexmedetomidine (9), and magnesium sulfate (10). Each carries specific limitations in children: nalbuphine requires a 2–3-minute onset period that may heighten anxiety in uncooperative patients; magnesium sulfate necessitates plasma-level monitoring and causes injection-site pain; and dexmedetomidine requires a 10-minute pretreatment window and risks bradycardia and hypotension.

Nalmefene hydrochloride is a selective μ-opioid receptor antagonist that may be particularly suited to SIC prevention. At low doses, nalmefene occupies μ-opioid receptors on tracheal mucosal epithelium and airway vagal afferent C-fiber terminals, which activate rapidly adapting receptors (RARs) and relay tussigenic signals to the medullary cough center (11, 12). Favorable compartment-specific pharmacokinetics—rather than intrinsic receptor subtype selectivity—may allow peripheral airway receptor occupancy to attenuate tussigenic signaling while preserving supraspinal analgesia (13). In adults undergoing breast surgery, nalmefene 0.1 μg/kg intravenously was effective in preventing SIC (12). However, this dose corresponds to the pediatric dose for reversing postoperative opioid overdose, raising concern that it may antagonize analgesia in children.

The primary objective of this study was to determine the ED50 of intravenous nalmefene for preventing SIC induced by sufentanil 0.2 μg/kg in children aged 1–6 years, and to assess dose-dependent effects on hemodynamic stability and postoperative pain.

## Methods

### Ethical approval and trial registration

This study was approved by the Institutional Review Board of Beijing Children's Hospital, National Center for Children's Health (Approval No. ([2025])-Y-006-D; 20 February 2025). The trial was prospectively registered with the Chinese Clinical Trial Registry (ChiCTR2500111207) on 25 February 2025, five days before first patient enrollment on 1 March 2025. Written informed consent was obtained from each parent or legal guardian before enrollment. The study was conducted in accordance with the CONSORT guidelines (14) and the Declaration of Helsinki (revised 2013) at Beijing Children's Hospital from 1 March to 30 June 2025.

### Study design and participants

This was a prospective, randomized, double-blind, placebo-controlled, dose-finding trial. Eligible patients were children aged 1–6 years with ASA physical status I–II, scheduled for elective laparoscopic cryptorchidism repair or laparoscopic high ligation of hernia sac under general anesthesia (anticipated procedure duration 1–2 h). Exclusion criteria were: (1) recent upper respiratory tract infection (within two weeks); (2) history of chronic cough, reactive airway disease, or asthma, or current use of bronchodilators or corticosteroids; (3) chronic opioid therapy; (4) renal or hepatic dysfunction; (5) known hypersensitivity to nalmefene, antitussive agents, or angiotensin-converting enzyme (ACE) inhibitors; and (6) pre-existing elevated intracranial, intraocular, or intra-abdominal pressure.

### Randomization and blinding

Participants were randomly allocated in a 1:1:1:1 ratio to Group A (normal saline placebo, 0 μg/kg), Group B (nalmefene 0.025 μg/kg), Group C (nalmefene 0.05 μg/kg), or Group D (nalmefene 0.1 μg/kg) using a computer-generated randomization sequence with permuted blocks of size 8, stratified by procedure type (hernia vs. cryptorchidism). Allocation concealment was maintained by having pharmacy personnel not involved in clinical care prepare all study syringes to a standardized volume of 1 mL per 10 kg body weight in sequentially numbered, sealed, opaque envelopes. The attending anesthesiologist and a dedicated outcome assessor (an experienced anesthesia nurse who counted coughs during the 1-minute observation window and was unaware of group allocation) were blinded to treatment assignment throughout the study.

### Anesthetic management

No preoperative sedation was administered. Standard monitoring was established upon arrival in the operating room: electrocardiography, non-invasive arterial blood pressure, and pulse oximetry (SpO2). Intravenous access was secured via a 22-gauge cannula in the dorsum of the hand. The study drug (nalmefene hydrochloride injection, 0.1 mg/mL; Yichang Humanwell Pharmaceutical Co., Ltd., China; or matched normal saline placebo) was administered intravenously over 5 s, followed by a 1-minute pretreatment interval. Sufentanil 0.2 μg/kg was then administered intravenously over 5 s. One minute after sufentanil injection, anesthesia was deepened with propofol 3 mg/kg and rocuronium 0.6 mg/kg, followed by insertion of an appropriately sized laryngeal mask airway (LMA; size 1.5 for body weight < 10 kg, 2.0 for 10–20 kg, 2.5 for 20–30 kg, and 3.0 for > 30 kg) and initiation of mechanical ventilation. Tidal volume and respiratory rate were adjusted to maintain end-tidal CO2 within 35–45 mmHg. Anesthesia was maintained with propofol and remifentanil, titrated to a bispectral index (BIS) of 40–60. Patients were transferred to the post-anesthesia care unit (PACU) on completion of surgery, and the LMA was removed after confirmation of adequate spontaneous ventilation (tidal volume > 6 mL/kg) and return of consciousness.

### Outcome measures

#### Primary outcome

The incidence of SIC, defined as the occurrence of one or more coughs within one minute of sufentanil injection, counted by a dedicated outcome assessor blinded to group allocation. The 1-minute observation window is consistent with prior SIC studies (1, 2) and reflects the peak tussigenic effect of intravenous sufentanil, which typically occurs within 30–60 s of bolus administration. The 1-minute pretreatment interval between study drug and sufentanil was selected on the basis that intravenous nalmefene reaches near-peak plasma concentrations within 1–2 min of administration (12), ensuring adequate receptor occupancy before sufentanil-induced tussigenic signaling is initiated.

#### Secondary outcomes

(1) SIC severity, graded on a validated four-point scale: grade 0, no cough; grade 1 (mild), 1–2 coughs; grade 2 (moderate), 3–4 coughs; grade 3 (severe), ≥5 coughs (15). (2) Hemodynamic parameters—mean arterial pressure (MAP), heart rate (HR), and SpO2—recorded at four time points: T0 (baseline), T1 (immediately after study drug), T2 (1 min after study drug), and T3 (1 min after sufentanil). Hemodynamic events were defined as follows: hypotension, MAP decrease > 20% from baseline; hypertension, MAP increase > 20%; tachycardia, HR increase > 20%; bradycardia, HR decrease > 20%. A decrease in SpO2 was defined as any fall below the T0 value; clinically significant desaturation was pre-specified as SpO2 < 95% or an absolute decrease ≥ 3 percentage points from baseline, and is reported separately. (3) Postoperative pain assessed by a PACU nurse blinded to group allocation using the Face, Legs, Activity, Cry, Consolability (FLACC) scale (16), performed approximately 5 min before the patient met PACU discharge criteria (Aldrete score ≥ 9).

### Sample size calculation

Sample size was calculated *a priori* using PASS 11.0 software. Pilot data indicated a baseline SIC incidence of 47.4% (9/19 patients) and a predicted incidence of 15.0% (6/40 patients) with nalmefene 0.05 μg/kg. To achieve 80% power at a two-sided *α* of 0.05 with an anticipated 10% dropout rate, 63 patients per group were required (total enrollment target: 252). The observed SIC incidence in Group C (30.2%) was higher than the pilot prediction (15.0%), reflecting the small pilot sample (*n* = 40). A *post-hoc* power analysis using observed incidences of 45.9% (Group A, *n* = 61) versus 11.3% (Group D, *n* = 62) confirmed that the study was adequately powered (> 99%) for the primary comparison. The implications of this discrepancy for dose-response precision are discussed in the Limitations section.

### Statistical analysis

Continuous variables are reported as mean ± standard deviation (SD) when normally distributed, or median (interquartile range, IQR) when normality was not met (Shapiro–Wilk test, *P* < 0.05), and were compared using one-way ANOVA or Kruskal–Wallis test, respectively. Categorical variables are reported as frequency (percentage) and compared using the Pearson chi-square or Fisher's exact test as appropriate. For contingency tables in which more than 20% of expected cell counts were below 5, a permutation-based chi-square (100,000 permutations) was used as the primary test. Pairwise *post-hoc* comparisons applied the Bonferroni correction. Monotonic dose-dependent trends across the four ordered groups were assessed by the Cochran-Armitage trend test, with dose scores set to the actual administered doses (0, 0.025, 0.05, and 0.1 μg/kg); the Z statistic and trend chi-square (*χ*^2^ trend) are reported alongside the overall *P* value. For the primary comparison (Group A versus Group D), treatment effects are reported as absolute risk reduction (ARR) with 95% CI (Newcombe method), relative risk (RR), odds ratio (OR), and number needed to treat (NNT). Missing hemodynamic values were handled by complete-case analysis; effective sample sizes are provided in the Table 3 footnote. All tests were two-sided; *P* < 0.05 was considered significant. Secondary outcomes were pre-specified as exploratory; no correction for multiplicity across secondary endpoints was applied, and all secondary *P* values should be interpreted with appropriate caution. Analyses were performed using SPSS Statistics 26.0 (IBM Corp., Armonk, NY, USA) and Python 3.x (scipy.stats; scipy.optimize; statsmodels).

The dose-response relationship was characterized by fitting a three-parameter sigmoidal Emax model with Emin constrained to zero:

E=Emax/[1+(D/ED50)n]

where E is the predicted SIC incidence at dose D (μg/kg); Emax is the maximum cough incidence; *n* is the Hill coefficient; and ED50 is the dose producing a 50% reduction in Emax. Constraining Emin to zero is physiologically justified because complete abolition of the cough reflex is theoretically achievable at sufficiently high antagonist concentrations, and the incidence at the highest tested dose (11.3%) closely approached this asymptote. This parameterization yields one residual degree of freedom (four data points minus three free parameters), permitting a limited goodness-of-fit assessment (approximate *χ*^2^1). Fitting used a weighted nonlinear least-squares algorithm (weights proportional to group sample sizes). The ED50, ED95, Emax, and *n* are reported with 95% CIs derived from 5,000-resample binomial bootstrap. Fit quality was additionally assessed by comparing the three-parameter model with an unconstrained four-parameter model via the Akaike Information Criterion (AIC).

As a pre-specified sensitivity analysis, the ED50 was independently estimated using individual-level logistic regression of the binary SIC outcome on nalmefene dose (*n* = 249 patients), defining the ED50 as the dose at which the predicted SIC incidence equals half the model-estimated placebo incidence. Concordance between the Emax model and logistic regression estimates was used to assess the robustness of the primary ED50 estimate.

## Results

### Patient flow and baseline characteristics

**Figure 1:**
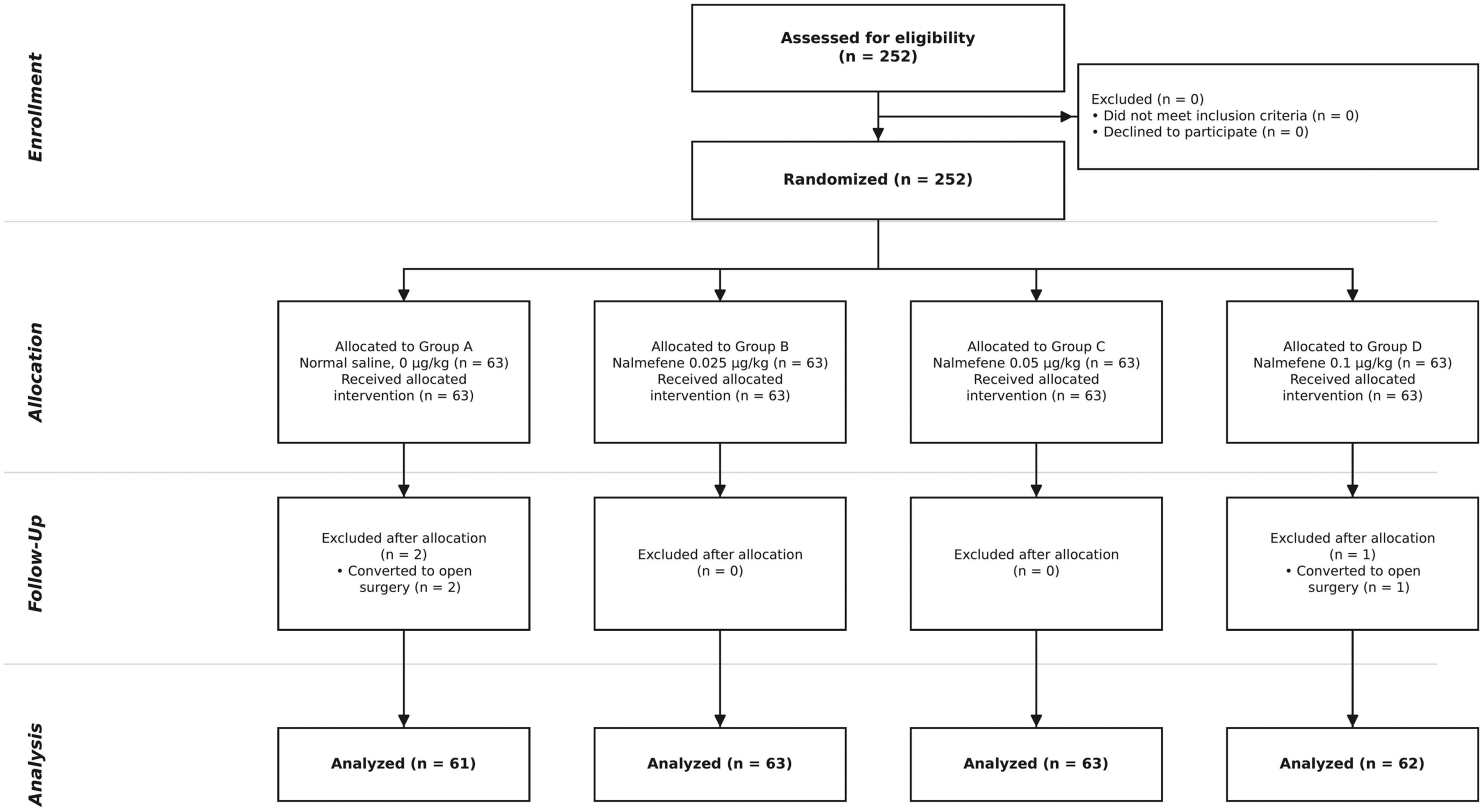
CONSORT flow diagram of 252 patients assessed for eligibility, all were randomized and allocated equally to four groups. Three patients were excluded after allocation due to unplanned conversion to open surgery (two in Group A, one in Group D), leaving 249 patients in the final analysis.

Two hundred and fifty-two patients were assessed for eligibility; all met inclusion criteria and were randomized. Three patients were excluded after allocation: two in Group A and one in Group D underwent unplanned conversion to open surgery due to intraoperative discovery of laparoscopic adhesions, approximately 10 min after study drug administration. Critically, the 1-minute SIC observation window had been completed in all three patients before conversion occurred; SIC was not observed in any of these patients. The final analysis therefore included 249 patients (Group A *n* = 61, Group B *n* = 63, Group C *n* = 63, Group D *n* = 62; Figure 1) and constitutes a per-protocol population. An intention-to-treat (ITT) sensitivity analysis incorporating all 252 randomized patients (assuming no SIC in the three excluded patients, consistent with the recorded data) yielded identical primary results and is not presented separately. Baseline characteristics—including age, sex, weight, ASA status, operative duration, and procedure type—were well balanced across groups (all *P* > 0.05; Table 1). The study population was predominantly male (72.1%, 76.2%, 84.1%, and 67.7% in Groups A–D, respectively), reflecting the sex distribution of the eligible procedures.

**Table 1:** Baseline demographic and clinical characteristics.

Characteristic	Group A (*n* = 61)	Group B (*n* = 63)	Group C (*n* = 63)	Group D (*n* = 62)	*P* value
Age (years)	4 (2.75–5.0)	3.5 (2–5.0)	3 (2.25–4.0)	3 (2–5.0)	0.928†
Sex (M/F)	44/17	48/15	53/10	42/20	0.182
Weight (kg)	16.66 ± 4.17	16.24 ± 4.92	16.15 ± 3.77	15.46 ± 4.28	0.652
ASA physical status (I/II)	61/0	63/0	63/0	62/0	1.000
Baseline SpO₂ (%)†	99 (98–100)	99 (98–100)	99 (98–99)	99 (98–100)	0.724
Operative duration (min)†	27.0 (15.0–59.0)	30.0 (20.0–49.0)	30.0 (20.0–56.0)	29.5 (18.2–69.8)	0.800
Procedure (hernia/cryptorchidism)	25/36	31/32	36/27	27/35	0.279

Values are median (IQR), mean ± SD, or n/n as appropriate. †Age, baseline SpO₂, and operative duration were non-normally distributed (Shapiro–Wilk *P* < 0.001 in all groups) and are presented as median (IQR), compared by Kruskal–Wallis test. Weight was normally distributed, presented as mean ± SD, and compared by one-way ANOVA. Categorical variables were compared by Pearson chi-square test. Operative duration data were available for *n* = 61, 61, 59, and 62 patients in Groups A–D, respectively (6 records missing from Groups B [*n* = 2] and C [*n* = 4]; complete-case analysis). ASA: American Society of Anesthesiologists; IQR: interquartile range; SD: standard deviation; SpO₂: peripheral oxygen saturation.

### Primary outcome: SIC incidence

SIC incidence decreased dose-dependently: 45.9% (28/61) in Group A, 38.1% (24/63) in Group B, 30.2% (19/63) in Group C, and 11.3% (7/62) in Group D (Table 2; overall *P* < 0.001). The Cochran-Armitage trend test confirmed a significant monotonic decrease (Z = −4.350, *χ*^2^ trend = 18.926, *P* < 0.001). *post-hoc* Bonferroni-corrected pairwise comparisons identified significant differences between Groups A and D (Bonferroni *P* = 0.0001) and between Groups B and D (Bonferroni *P* = 0.005). Group B (38.1%) was not significantly different from Group A (Bonferroni *P* = 1.000), indicating that the 0.025 μg/kg dose falls below the effective threshold for SIC prevention. No other pair reached significance. For the primary comparison (Group D versus Group A), the ARR was 34.6% (95% CI: 18.9%–48.2%), the RR was 0.25 (95% CI: 0.12–0.52), the OR was 0.15 (95% CI: 0.06–0.38), and the NNT was 2.9.

**Table 2:** Incidence and severity of sufentanil-induced cough, SpO₂ changes, and postoperative pain by group.

Variable	Group A (*n* = 61)	Group B (*n* = 63)	Group C (*n* = 63)	Group D (*n* = 62)	*P* value	*P* trend
SIC incidence, *n* (%)	28 (45.9)	24 (38.1)	19 (30.2)	7 (11.3)^a^	< 0.001	< 0.001
SIC severity, *n* (%)					< 0.001	
None	33 (54.1)	39 (61.9)	44 (69.8)^a^	55 (88.7)^a^		
Mild (1–2 coughs)	6 (9.8)	14 (22.2)	10 (15.9)	4 (6.5)		
Moderate (3–4 coughs)	12 (19.7)	8 (12.7)	7 (11.1)	3 (4.8)		
Severe (≥5 coughs)	10 (16.4)	2 (3.2)	2 (3.2)	0 (0.0)^a^		0.0005
SpO₂ decrease (any), *n* (%)	18 (29.5)	21 (33.3)	16 (25.4)	11 (17.7)	0.233	0.066
SpO₂ decrease (clinically significant), *n* (%)	3 (4.9)	4 (6.3)	3 (4.8)	2 (3.2)	0.887	0.283
FLACC score, *n* (%)						
0	55 (90.2)	59 (93.7)	59 (93.7)	57 (91.9)	0.267^b^	
1–3	3 (4.9)	1 (1.6)	1 (1.6)	5 (8.1)		
4–6	3 (4.9)	3 (4.8)	3 (4.8)	0 (0.0)		
7–10	0 (0.0)	0 (0.0)	0 (0.0)	0 (0.0)		

a *P* < 0.05 versus Group A (post-hoc Bonferroni correction).

b Overall FLACC *P* = 0.267 by permutation chi-square (100,000 permutations); asymptotic chi-square *P* = 0.273; binary FLACC (score 0 vs. > 0) *P* = 0.866; Cochran-Armitage trend *P* = 0.827. Permutation test applied because 8 of 12 expected cell counts were < 5. Clinically significant SpO₂ decrease: SpO₂ < 95% or absolute decrease ≥ 3 percentage points from baseline.

FLACC, Face, Legs, Activity, Cry, Consolability; SIC, sufentanil-induced cough; SpO₂, peripheral oxygen saturation.

### Secondary outcomes

#### SIC severity

Nalmefene reduced SIC severity in a dose-dependent manner (Table 2). In Group A, 10 patients (16.4%) experienced severe cough (≥ 5 episodes) and 12 (19.7%) experienced moderate cough (3–4 episodes). In Group D, no patient experienced severe cough and only 3 (4.8%) had moderate cough. The incidence of severe cough was significantly lower in Group D than in Group A (Fisher's exact *P* = 0.001; Cochran-Armitage: Z = −3.469, *χ*^2^ trend = 12.034, *P* = 0.0005).

#### Hemodynamic parameters

The incidences of tachycardia/bradycardia and hypotension/hypertension did not differ significantly across groups at any time point (all *P* > 0.05; Table 3). Hemodynamic monitoring data were incomplete for a small proportion of patients, most notably at T3 in Group A (blood pressure: 54/61 available; heart rate: 56/61 available). These missing records were attributable to monitoring interruptions during SIC episodes, when immediate airway repositioning precluded reliable non-invasive blood pressure and electrocardiographic capture. To assess the robustness of this finding, a worst-case sensitivity analysis was performed: imputing hypotension for all 7 patients with missing T3 blood pressure in Group A yielded an estimated hypotension rate of 44.3% (27/61), which remained non-significantly different from Group D (37.1%, 23/62; *P* = 0.392). The overall missing rate was 2.5% (57/2,241 observations across 249 patients   ×   3 hemodynamic variables   ×   3 time points); all proportions were calculated on available data.

**Table 3:** Incidence of intraoperative hemodynamic events by group and time point.

Variable	Time	Group A	Group B	Group C	Group D	*P* value	*P* trend
Tachycardia or bradycardia, *n* (%)	T1	16 (26.7)	26 (41.3)	21 (34.4)	16 (25.8)	0.210	0.539
	T2	27 (45.8)	31 (49.2)	21 (34.4)	23 (37.1)	0.292	0.220
	T3	31 (55.4)	32 (51.6)	23 (37.7)	27 (45.0)	0.231	0.287
Hypotension or hypertension, *n* (%)	T1	16 (27.1)	17 (27.0)	26 (41.3)	19 (31.1)	0.273	0.452
	T2	25 (43.1)	21 (33.3)	23 (36.5)	27 (45.0)	0.510	0.564
	T3	20 (37.0)	24 (38.7)	21 (34.4)	23 (39.7)	0.939	0.541

T1: immediately after study drug; T2: 1 min after study drug; T3: 1 min after sufentanil. Tachycardia/bradycardia: HR change > 20% from baseline. Hypotension/hypertension: MAP change > 20% from baseline. Group headers reflect total randomized n; all proportions are based on available data. Effective sample sizes — Tachycardia or bradycardia: T1 (A *n* = 60, B *n* = 63, C *n* = 61, D *n* = 62), T2 (A *n* = 59, B *n* = 63, C *n* = 61, D *n* = 62), T3 (A *n* = 56, B *n* = 62, C *n* = 61, D *n* = 60). Hypotension or hypertension: T1 (A *n* = 59, B *n* = 63, C *n* = 63, D *n* = 61), T2 (A *n* = 58, B *n* = 63, C *n* = 63, D *n* = 60), T3 (A *n* = 54, B *n* = 62, C *n* = 61, D *n* = 58).

HR, heart rate; MAP, mean arterial pressure.

#### Oxygen saturation

Any SpO2 decrease (below T0 baseline) occurred in 29.5%, 33.3%, 25.4%, and 17.7% of patients in Groups A–D, respectively (*P* = 0.233; Table 2). The incidence of clinically significant desaturation (SpO2 < 95% or absolute decrease ≥ 3 percentage points) was 4.9%, 6.3%, 4.8%, and 3.2% (*P* = 0.887). A numerically decreasing trend in any SpO2 decrease did not reach significance (Cochran-Armitage: Z = −1.836, *χ*^2^ trend = 3.371, *P* = 0.066).

#### Postoperative pain

FLACC scores were comparable across groups (permutation chi-square *P* = 0.267; Table 2). Median FLACC scores were 0 (IQR 0–0) in all groups. The FLACC > 0 rate was 9.8%, 6.3%, 6.3%, and 8.1% in Groups A–D (binary chi-square *P* = 0.866; Cochran-Armitage trend *P* = 0.827), with Group D (8.1%) numerically lower than Group A (9.8%). Given that 8 of 12 expected cells in the three-category table fell below 5, the permutation-based chi-square is reported as the primary *P* value.

#### Adverse events

No adverse events—including respiratory depression, nausea, vomiting, or allergic reactions—were recorded in any patient.

### Dose-response analysis and ED50

**Figure 2:**
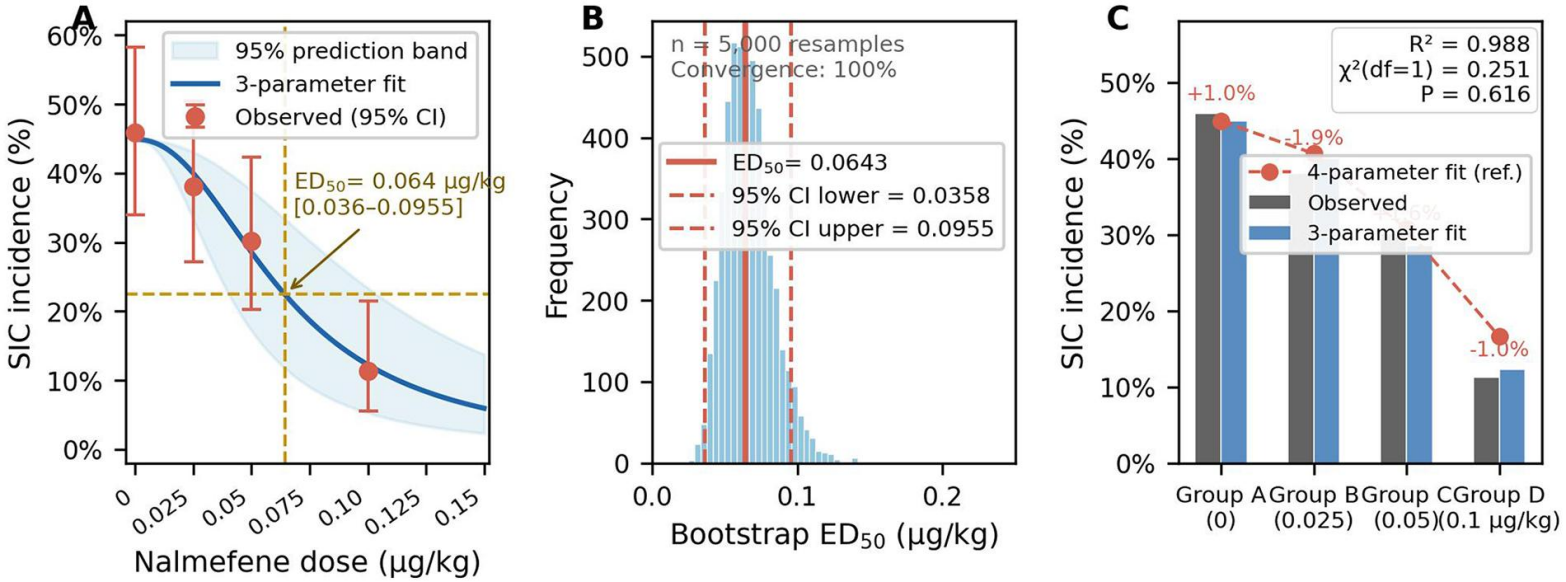
Dose–response analysis of nalmefene for the prevention of sufentanil-induced cough (SIC). **(A)** Dose–response relationship between intravenous nalmefene dose and SIC incidence. Filled circles represent the observed SIC incidence at each dose (0, 0.025, 0.05, and 0.1 µg/kg); error bars denote 95% binomial confidence intervals (CIs). The solid line shows the fitted three-parameter sigmoidal E_max_ model (E_min_ fixed at 0), and the shaded band represents the 95% bootstrap prediction interval. Model parameters (5,000-resample binomial bootstrap) were as follows: E_max_ = 0.449 (95% CI, 0.412–0.491); Hill coefficient, *n* = 2.22 (95% CI, 1.08–3.96); ED_50_ = 0.064 µg/kg (95% CI, 0.036–0.096 µg/kg); weighted R^2^ = 0.988; χ^2^_1_ = 0.251 (*P* = 0.616); ΔAIC versus the four-parameter model = 0.446. The dashed vertical line marks the ED_50_ point estimate. The corresponding ED_95_ (0.242 µg/kg; 95% CI, 0.114–1.948 µg/kg) required extrapolation beyond the highest tested dose and should therefore be interpreted with caution. **(B)** Bootstrap distribution of the ED_50_ estimate. The sampling distribution of ED_50_ was derived from 5,000 binomial bootstrap resamples (convergence rate, 100%). The solid red line indicates the point estimate (ED_50_ = 0.0643 µg/kg), and the dashed red lines denote the lower (0.0358 µg/kg) and upper (0.0955 µg/kg) bounds of the 95% CI. The distribution was approximately symmetric and tightly clustered, indicating a robust and stable estimate. **(C)** Comparison of observed and model-predicted SIC incidence across dose groups. Observed SIC incidence (grey bars) is compared with the three-parameter model fit (blue bars) for each group (Group A, 0; Group B, 0.025; Group C, 0.05; Group D, 0.1 µg/kg). The dashed red line connecting filled circles denotes the four-parameter model fit, shown as a reference. Values above each pair of bars indicate the residual between the observed and three-parameter fitted incidences (+1.0%, −1.9%, +1.6%, and −1.0%). The three-parameter model showed excellent goodness of fit (*R*^2^ = 0.988; χ^2^_1_ = 0.251; *P* = 0.616), with no significant departure from the observed data. Abbreviations: SIC, sufentanil-induced cough; CI, confidence interval; ED_50_/ED_95_, dose producing 50%/95% of the maximal effect; E_max_, maximal effect; E_min_, minimal effect; AIC, Akaike information criterion.

The three-parameter Emax model provided an adequate fit to the observed incidence data (weighted R^2^ = 0.988; approximate *χ*^2^1 = 0.251, *P* = 0.616; Figure 2). The unconstrained four-parameter model offered no meaningful improvement (*Δ*AIC = 0.446 < 2); the three-parameter model was therefore retained as the primary model. Estimated parameters (5,000-resample bootstrap) were: Emax = 0.449 (95% CI: 0.412–0.491), closely approximating the observed incidence in Group A (45.9%); Hill coefficient *n* = 2.22 (95% CI: 1.08–3.96), indicating a moderately steep dose-response relationship, though with wide uncertainty given the four-point curve; and ED50 = 0.064 μg/kg (95% CI: 0.036–0.096 μg/kg). The individual-level logistic regression sensitivity analysis yielded a concordant ED50 estimate of 0.062 μg/kg, confirming the robustness of the primary estimate. The estimated ED95 was 0.242 μg/kg (95% CI: 0.114–1.948 μg/kg); this value requires substantial extrapolation beyond the highest tested dose (0.1 μg/kg) and should be regarded as exploratory.

## Discussion

In this dose-finding randomized controlled trial, intravenous nalmefene reduced SIC incidence dose-dependently in children aged 1–6 years, with an ED50 of 0.064 μg/kg (95% CI: 0.036–0.096 μg/kg). Nalmefene 0.1 μg/kg reduced absolute SIC incidence by 34.6 percentage points (45.9% to 11.3%) relative to saline (Bonferroni *P* = 0.0001; RR = 0.25; NNT = 2.9), meaning that approximately 3 children need to be treated at this dose to prevent one SIC episode. Notably, 0.025 μg/kg (Group B) did not significantly reduce SIC incidence compared with placebo (Bonferroni *P* = 1.000), identifying this dose as sub-threshold and supporting the clinical relevance of the ED50 estimate. Hemodynamic stability was preserved at all doses, and postoperative FLACC scores did not increase at any dose, including the highest tested. The individual-level logistic regression sensitivity analysis yielded a concordant ED50 of 0.062 μg/kg, confirming the primary Emax model estimate. These findings identify 0.064 μg/kg as a clinically applicable ED50 for SIC prevention in this age group.

The mechanism underlying nalmefene's antitussive effect likely reflects compartment-specific pharmacokinetics rather than intrinsic receptor subtype selectivity. SIC is primarily mediated by sufentanil acting on μ-opioid receptors expressed on tracheal mucosal epithelium and airway vagal afferent C-fiber terminals, which activate rapidly adapting receptors and relay tussigenic signals to the medullary cough center (11, 12). At the low doses studied, nalmefene has been proposed to preferentially reach peripheral airway μ-opioid receptors before achieving substantial supraspinal concentrations, thereby attenuating the tussigenic signaling of sufentanil while preserving central analgesia—a differential receptor occupancy driven by pharmacokinetic rather than pharmacodynamic factors. The estimated Hill coefficient of 2.22 (95% CI: 1.08–3.96) is consistent with concentration-dependent competitive occupancy at peripheral airway receptor sites, although its wide confidence interval reflects the inherent uncertainty of estimating this parameter from only four group-level data points.

The clinical significance of SIC prevention is amplified in young children by their limited oxygen reserves. Although no statistically significant between-group difference was detected in any SpO2 measure, the numerically lower rate of clinically significant desaturation in Group D (3.2% versus 4.9% in Group A) is directionally consistent with clinical benefit. Future studies with larger sample sizes may demonstrate a statistically significant protective effect on oxygenation.

Postoperative analgesia was preserved across all dose groups. The FLACC > 0 rate in Group D (8.1%) was numerically lower than in Group A (9.8%), and no statistically significant between-group differences were detected at any level of analysis (binary FLACC *P* = 0.866; trend *P* = 0.827). This finding is consistent with Xie et al. (12), who reported no significant increase in postoperative pain with nalmefene 0.1 μg/kg in adults. Collectively, these results support the analgesic safety of the ED50 dose of 0.064 μg/kg for clinical use.

Compared with other preventive strategies, nalmefene offers several practical advantages. Unlike nalbuphine (0.3 mg/kg) (7), nalmefene does not require a 2–3-minute onset period that may increase anxiety in uncooperative children. Unlike magnesium sulfate (10), it requires no plasma-level monitoring and causes no injection-site discomfort. Unlike dexmedetomidine (9), it does not require a 10-minute pretreatment window and does not risk hemodynamic instability. Remifentanil effectively suppresses SIC (8) but adds a second opioid to the induction sequence, complicating dose titration.

Several limitations warrant consideration. First, this was a single-center study, and external validation is required before generalizing the ED50 estimate. Second, only children aged 1–6 years undergoing two specific laparoscopic procedures were studied; the ED50 in infants, older children, or those undergoing other procedures remains unknown. Third, the study population was predominantly male (68%–84% across groups), reflecting the sex distribution of the eligible procedures; applicability to female children or populations with balanced sex distribution needs verification. Fourth, the dose-response model used four group-level data points with one residual degree of freedom, limiting parameter precision; the wide confidence intervals for ED50 and ED95 reflect genuine pharmacodynamic uncertainty. Fifth, only the sufentanil 0.2 μg/kg dose was evaluated. Sixth, the observed SIC incidence in Group C (30.2%) substantially exceeded the pilot-predicted value (15.0%), attributable to the small pilot cohort; a *post-hoc* power analysis confirmed adequate power (> 99%) for the primary comparison, but precision of intermediate-dose estimates was likely reduced. Finally, a fixed-dose parallel design—rather than a sequential up-and-down method—was chosen to allow simultaneous evaluation of safety across the full dose range under rigorously blinded conditions. Future multicenter trials employing sequential allocation designs would provide more precise ED50 and ED95 estimates with fewer patients.

## Conclusions

Intravenous nalmefene pretreatment reduces the incidence and severity of SIC during anesthetic induction in children aged 1–6 years in a dose-dependent manner, without significant perturbation of hemodynamic stability or postoperative analgesia. The ED50 is 0.064 μg/kg (95% CI: 0.036–0.096 μg/kg). In the absence of prophylaxis, the SIC rate was 45.9%, underscoring the clinical importance of routine prevention in this age group. Nalmefene represents a promising, well-tolerated option for SIC prevention in pediatric anesthetic practice.

## Data Availability

The raw data supporting the conclusions of this article will be made available by the authors, without undue reservation.
